# Supplementary material for: Site-specific monoubiquitination downregulates Rab5 by disrupting effector binding and guanine nucleotide conversion
Source: eLife. 2017 Oct 2;6:e29154. doi: 10.7554/eLife.29154 (PMC5624781; doi:10.7554/eLife.29154)
Supplement: Transparent reporting form [file elife-29154-transrepform.docx]

***eLife’s* transparent reporting form**

We encourage authors to provide detailed information *within their submission* to facilitate the interpretation and replication of experiments. If you have any questions, please contact us: [editorial@elifesciences.org](mailto:editorial@elifesciences.org).

**Sample-size estimation**

- You should state whether an appropriate sample size was computed when the study was being designed
- You should state the statistical method of sample size computation and any required assumptions
- If no explicit power analysis was used, you should describe how you decided what sample (replicate) size (number) to use

Please outline where this information can be found within the submission (e.g., page numbers or figure legends), or explain why this information doesn’t apply to your submission:

Our manuscript does not have sample-size estimation data.

**Replicates**

- You should report how often each experiment was performed
- You should include a definition of biological versus technical replication
- The data obtained should be provided and sufficient information should be provided to indicate the number of independent biological and/or technical replicates
- If you encountered any outliers, you should describe how these were handled
- Criteria for exclusion/inclusion of data should be clearly stated
- High-throughput sequence data should be uploaded before submission, with a private link for reviewers provided (these are available from both GEO and ArrayExpress)

Please outline where this information can be found within the submission (e.g., page numbers or figure legends), or explain why this information doesn’t apply to your submission:

1. Ubiquitination assay/ Cytosol/membrane fractionation assay/ EGF induced EGFR degradation assay

Information for the replicates was written in material and methods/Figure legends. (Page 27, line 526, Page 28, line 542 and line 551).

2. GEF mediated GDP release and GTP loading assay

Information for the replicates was written in material and methods/Figure legends. (Page 30, line 589).

3. Effector protein binding assay by bio-layer interferometry (BLI)

Information for the replicates was written in material and methods (Page 30, line 596-597)

**Statistical reporting**

- Statistical analysis methods should be described and justified
- Raw data should be presented in figures whenever informative to do so (typically when N per group is less than 10)
- For each experiment, you should identify the statistical tests used, exact values of N, definitions of center, methods of multiple test correction, and dispersion and precision measures (e.g., mean, median, SD, SEM, confidence intervals; and, for the major substantive results, a measure of effect size (e.g., Pearson's r, Cohen's d)
- Report exact p-values wherever possible alongside the summary statistics and 95% confidence intervals. These should be reported for all key questions and not only when the p-value is less than 0.05.

1. GEF mediated GDP release and GTP loading assay

Information for graph fitting and S.D. estimation is written in materials and methods (Page 30, line 589). Number of experiments with S.D. is also reported in (Figure 5, and Supplementary Table 3)

2. Effector protein binding assay by bio-layer interferometry (BLI)

Information for graph fitting and S.D. estimation is written in materials and methods (Page 30, line 597 – Page 31, 604). k_on_, k_off_, K_d_ values with S.E.M are reported in (Figure 6, and Supplementary Table 4)

Please outline where this information can be found within the submission (e.g., page numbers or figure legends), or explain why this information doesn’t apply to your submission:

(For large datasets, or papers with a very large number of statistical tests, you may upload a single table file with tests, Ns, etc., with reference to page numbers in the manuscript.)

**Additional data files (“source data”)**

- We encourage you to upload relevant additional data files, such as numerical data that are represented as a graph in a figure, or as a summary table
- Where provided, these should be in the most useful format, and they can be uploaded as “Source data” files linked to a main figure or table
- Include model definition files including the full list of parameters used
- Include code used for data analysis (e.g., R, MatLab)
- Avoid stating that data files are “available upon request”

Please indicate the figures or tables for which source data files have been provided:

Not applicable.
